# Poly(butylene adipate-*co*-terephthalate)/Graphene Oxide/Carbon Black Nanocomposite Films for Agricultural Applications: From Mechanical Performance to Ecotoxicological Effects

**DOI:** 10.1021/acsomega.5c05280

**Published:** 2025-08-13

**Authors:** Patrícia M.S. Souza, Rosa Maria Vercelino Alves, Maria Aparecida Marin-Morales, Guilhermino J. M. Fechine

**Affiliations:** † Engineering School, Mackenzie Presbyterian University, Rua da Consolação, 896, São Paulo, São Paulo 01302-907, Brazil; ‡ Mackenzie Institute for Research in Graphene and Nanotechnologies − MackGraphe, Mackenzie Presbyterian University, Rua da Consolação, 896, São Paulo, São Paulo 01302-907, Brazil; § Packaging Technology Center, 218464 Institute of Food Technology, Av. Brasil, 2880, Campinas, São Paulo 13070-178, Brazil; ∥ Department of Biology, Institute of Biosciences, São Paulo State University (UNESP), Av. 24-A, 1515, Rio Claro, São Paulo 13506-900, Brazil

## Abstract

Poly­(butylene adipate-*co*-terephthalate) (PBAT) nanocomposite films containing graphene oxide (GO) and carbon black (CB) were prepared through plane extrusion to assess their potential for agricultural applications. The films with 0.25% and 0.50% w/w of GO content and 1.5% w/w of CB and combinations of both fillers at the specified concentrations were characterized. CB effectively blocked UV/vis transmittance, and its combination with GO at a lower concentration resulted in satisfactory mechanical properties for mulch film applications. The formulation containing only GO (0.25%) yielded favorable results in elongation and tenacity for mulch film applications, along with effective UV/vis transmittance blocking, although not sufficient to prevent weed growth, making this film more suitable for greenhouse applications than for mulch films. In both scenarios, the presence of the fillers did not affect the biodegradation behavior of PBAT in soil, and none of the tested films exhibited any ecotoxicological effects on *Allium cepa*, highlighting the potential of the studied formulations as biodegradable and environmentally friendly agricultural films, thus encouraging further exploration in this area.

## Graphical abstract

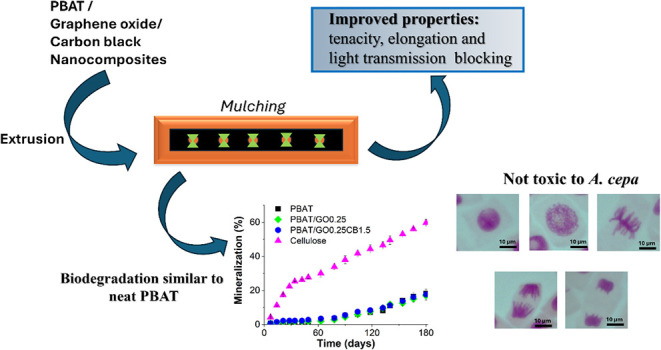

## 1 Introduction

Several studies have explored the potential of the polymer poly­(butylene adipate-*co*-terephthalate) or PBAT as an alternative to conventional plastics across various applications, aiming to reduce the waste sent to landfills.^1–3^ This polymer is currently commercially available in Germany, Italy, and China from companies like BASF, NOVAMONT, KINGFA, TUNHE, XINFU, and JINHUI, with production levels ranging from 20 000 to 60, 000 tons per year.^4^

PBAT is a copolyester derived from fossil resources, and its production relies on the polycondensation reaction of 1,4-butanediol, adipic acid, and terephthalic acid.^5^ This polymer holds potential for applications in agriculture, such as mulching films, which are commonly utilized to control weed growth, reduce evaporation, and minimize soil erosion.^6^ The use of biodegradable polymers in agriculture has allowed for landfill diversion. These films can degrade directly in the field after use, thereby avoiding the costs associated with removal and disposal.^7^ However, PBAT is highly sensitive to photodegradation, which compromises its mechanical properties during use, representing the main obstacle to the application of this polymer in agriculture.^8^

Various additives can reduce the photodegradation of PBAT. Carbon black is commonly used for this purpose and is also applied in mulch films to block photosynthetically active radiation (400–700 nm), which prevents weed growth.^2,7^ Carbon black (CB) primarily consists of finely divided, spherical carbon particles created by the incomplete combustion of carbonaceous fuels. It has a high surface-to-volume ratio due to its small size (usually below 50 nm).^9^ The literature suggests using carbon black to photostabilize PBAT, but in combination with different light stabilizers to enhance its effectiveness (phenolic/amine stabilizers and antioxidants).^10,11^

No studies in the literature were found using carbon black combined with graphene oxide for PBAT protection against UV. Graphene oxide (GO) is a two-dimensional material and is the oxidized form of graphene, featuring oxygen functional groups on the sp^2^ carbon basal plane.^12^ GO can function as an antioxidant, exhibiting free radical scavenging activity associated with the sp^2^ carbons on its basal surface.^13^ In addition, it has the potential to enhance the mechanical properties of polymers, such as elongation and tensile strength.^14,15^

Chah et al.^16^ emphasized that research from 1973 to 2022 on bioplastics reveals a significant lack of investigation into the ecological effects on soil properties, plant responses, soil biology, and toxicity. In this context, the bioassay using the *Allium cepa* test organism—an *in vivo* test that evaluates the cytotoxic, genotoxic, and mutagenic potential of various compounds and environmental mixtures—plays a crucial role.^17–20^ The main advantages of using this organism include its large size and small number of chromosomes, which facilitate damage observation by light microscopy. Additionally, it is cost-effective, requires small sample volumes for testing, and allows for direct exposure to samples of interest.^21^

In the field of polymers, the *A. cepa* bioassay has been utilized in two main approaches: investigating aqueous solutions of conventional microplastics, such as polystyrene and polypropylene,^22–24^ and analyzing aqueous extracts from soil/compost samples before and after the disintegration and biodegradation of bioplastics like poly­(lactic acid) and poly­(butylene adipate-*co*-terephthalate). Souza et al.^10^ noted the absence of toxic effects on *A. cepa* for aqueous extracts from soil containing PBAT films (with and without carbon black and other UV stabilizers) before and after biodegradation. However, the initial concentration of materials in the soil used by the authors was 0.2%. A more recent standard focusing on biodegradable mulch films recommends an initial concentration of 1%, which allows for the simulation of a scenario with repeated applications.^25^

In this work, the potential of PBAT/graphene oxide and carbon black nanocomposites was investigated. Various aspects were characterized, including morphology, mechanical properties, barrier properties, and UV/vis transmittance, to evaluate the potential of these materials as films for agriculture. Considering the end-of-life scenario of incorporation into soil, biodegradation, and ecotoxicity analysis using the *A. cepa* test as a bioassay were also conducted to assess the environmental impact.

The combination of carbon black with graphene oxide for agricultural applications proposed here is unprecedented, and conducting an assessment that covers everything from material properties to ecotoxicological effects is innovative. This is especially true because environmental safety assessments of biodegradable plastics represent one of the main knowledge gaps in this field.

## 2 Materials and Methods

### 2.1 Materials

The nanocomposites were prepared using poly­(butylene adipate-*co*-terephthalate) (PBAT) Ecoflex F Blend C1200 from BASF (density: 1.25–1.27 g/cm^3^; melt flow rate: 2.7–4.9 g/10 min), carbon black BP460 from CABOT (density: 375 kg/m^3^; iodine number: 82 mg/g; oil absorption number: 102 cc/100 g), and graphene oxide prepared with graphite powder supplied by Sigma-Aldrich (diameter <20 μm) with a lateral size of 520 ± 210 nm. Both carbon black and PBAT were commercially available, while graphene oxide was synthesized using a modified Hummers method in a bench reactor.^26^

### 2.2 Preparation of PBAT and PBAT/GO/Carbon Black Nanocomposite Films

Initially, the polymer PBAT pellets underwent cryogenic grinding in a knife mill. The polymer granules and GO suspension in water were mixed in a glass bowl on a heating plate to facilitate water evaporation. The mixture was heated to approximately 60 °C. The exfoliated GO suspension at a concentration of 1 mg/mL was added to PBAT to create the masterbatch (MB) with a 2% mass content of GO. This method of integrating GO into the polymer is referred to as Solid–Solid Deposition (SSD).^14^ The MB was processed using a Thermo Scientific twin-screw extruder, model Process 11 (L/D ratio = 40), at 100 rpm with a 4 g/min feed rate. The temperature profile employed was: 150 °C/165 °C/170 °C/170 °C/175 °C/175 °C/170 °C/170 °C from hopper to die. Subsequently, the PBAT granules were processed with MB on the same equipment and under identical conditions to obtain PBAT compositions with GO at the specified mass concentrations. This process was also performed for neat PBAT. Formulations with carbon black were prepared by incorporating this additive directly, in powder form, into the formulations at a rate of 1.5% by mass. Table 1 presents the nomenclature and composition of the prepared formulations.

**Table 1:** Formulations, Nomenclature, and Composition

Nomenclature	Graphene oxide—GO (% in mass)	Carbon black—CB (% in mass)
**PBAT**	-	-
**PBAT/GO0.25**	0.25	-
**PBAT/GO0.50**	0.50	-
**PBAT/CB1.5**	-	1.5
**PBAT/GO0.25CB1.5**	0.25	1.5
**PBAT/GO0.50CB1.5**	0.50	1.5

The GO concentrations (0.25 and 0.50% by weight) were discussed for packaging applications in a previous work.^27^ In this study, these formulations are mainly focused on their agricultural applications. The association with 1.5% by weight of carbon black was determined based on the literature, which shows that this is an optimal amount of this additive, considering PBAT UV protection.^2^

After processing, the extruded filaments were cryogenically pelletized due to PBAT’s high flexibility. To produce the films, the pellets of neat PBAT and nanocomposite materials were fed into a Thermo Scientific single-screw extruder, model Haake Rheomex OS (L/D ratio = 25), at a speed of 10 rpm, following this temperature profile: 150 °C, 155 °C, 160 °C, and 175 °C from the hopper to the die.

### 2.3 Characterization of Polymer Nanocomposites

The molecular weight was determined through viscometric analysis using a ball drop viscometer (Thermo Scientific, Type C), following ASTM D4603. Initially, solutions of PBAT pellets, neat PBAT films, and nanocomposite films were prepared at a concentration of 0.025 g/mL in chloroform with 40 mL of each solution utilized in the equipment. The intrinsic viscosity was calculated from the average of 10 measurements of relative viscosity, employing the Billmeyer equation (eq 1), followed by the calculation of molecular weight according to the Mark–Houwink–Sakurada equation (eq 2).

1 $$\left[n\right]=\frac{0,25\left(n_{r}-1+3\ln n_{r}\right)}{C}$$

Where:

η—intrinsic viscosity;

η_r_—relative viscosity (*t*/*t*_0_—where *t* is the flux time in the solution and *t*_0_ is the flux time in the solvent);

*C*—concentration of the polymeric solution (0.025 g/mL).

2 $$\left[n\right]=KM^{\alpha }$$

Where:

[η]—intrinsic viscosity (mL/g);

*M*—molecular weight;

*K* and α—constants considering PBAT–chloroform (*K* = 8.5 × 10^–3^; α = 0.76).^27^

Light transmission (%*T*) for UV and visible light of the PBAT and nanocomposite films was measured using a UV–visible spectrophotometer (UV-3600 Plus, Shimadzu) with an integrated sphere, a Δλ of 1 nm, and a speed of 2 nm/s. The films were scanned using a wavelength range of 220–800 nm in duplicate. The percentage of light transmission was calculated from the integrated area under the light transmission spectrum divided by the bandwidth, 100 nm for UV radiation (wavelength: 300–400 nm) and 400 nm for visible light (wavelength: 400–800 nm). The %*T* represents the ability of the filler to block UV and visible light.

X-ray diffraction was performed using a Rigaku Miniflex II diffractometer with Kα Cu radiation (λ = 0.154 nm) over an angular range of 2θ from 5° to 90° at a scanning speed of 10°/min. The degree of crystallinity (%) was calculated as the ratio of the crystalline peak area (*I*_c_) to the amorphous halo area (*I*_a_), as shown in eq 3

3 $$X_{c}=\frac{I_{c}}{I_{c}+I_{a}}\times 100$$

Thermogravimetric analysis (TGA) was conducted using the SDT Q600 model from TA Instruments, operated under a nitrogen atmosphere with a flow rate of 100 mL/min, ranging from 30 to 600 °C at a heating rate of 10 °C/min. The thermal behavior of the nanocomposites was examined using Differential Scanning Calorimetry (DSC) with DSC-60PLUS (Shimadzu) equipment, which also operated under a nitrogen atmosphere at a flow rate of 50 mL/min. The heating and cooling cycles occurred within the temperature range of 25–160 °C at 10 °C/min. The degree of crystallinity was calculated during both the first and second heating according to eq 4:

4 $$X_{c}=\frac{\Delta H_{f}}{\Delta H_{m}\left(1-w\right)}\times 100$$

Where:

△*H*_f_—enthalpy fusion;

△*H*_m_—enthalpy fusion of 100% crystalline PBAT (114 J/g);^28^

*w*—mass fraction of GO.

Optical Microscopy (Nikon H550L-Eclipse LV100ND) was used to analyze the particle distribution. ImageJ software was applied to quantify the surface area of 200 agglomerates per sample.

The films underwent cryogenic fracture in liquid nitrogen. A layer of gold was deposited on the fracture surfaces using a Bal-Tec metallizer, with an amperage of 22 mA for 150 s. The fracture surface was then observed through a JEOL microscope, model JSM-7800F, operating at 5 kV.

Tensile tests were conducted on the film materials following ASTM D882 ^29^ at a deformation rate of 500 mm/min. The equipment utilized was an Instron universal testing machine, model 5982, equipped with a 500 N load cell. The results obtained represent the average of five measurements. The tensile strength (σ), elongation at break (ε), and Young’s modulus (E) were calculated. The tenacity (the area under the σ–ε curves) was also determined. The specimens for this test were cut in the longitudinal direction of the extrusion.

The oxygen transmission rate (OTR) of films was measured using an Oxygen Permeation Analyzer (OX-TRAN 2/20, Mocon) with a coulometric sensor, following ASTM D3985,^30^ at 23 °C under dry conditions. The analyses were conducted in duplicate. The oxygen permeability coefficient (OP) was calculated using eq 5:

5 $$\mathrm{OP}=\mathrm{OTR}\left(\frac{l}{p1-p2}\right)$$

Where:

OP—the oxygen permeability coefficient (cm^3^ mil m^–2^ d^–1^ atm^–1^);

OTR—the oxygen transmission rate (cm^3^ m^–2^ d^–1^);

*l*—film thickness (mil);

*p*1 – *p*2—gradient of oxygen partial pressure (1 atm).

### 2.4 Biodegradation and Ecotoxicity Analysis

The biodegradation test was conducted according to the Brazilian standard NBR 14283, utilizing Bartha’s respirometric method for two selected best-performing formulations, considering agriculture applications based on previous analyses of nanocomposite films and also the neat PBAT film as a control. The biodegradation of PBAT and nanocomposite films was assessed in commercially available soil (Geolia). The soil was dried for 72 h at room temperature and sieved (<2 mm) before the test. The soil used in this test presents the following properties: total carbon: 11 000 mg/kg; total nitrogen: 622.9 mg/kg; C/N (Carbon/Nitrogen) ratio: 17.7 mg/kg; pH: 4.75; clay content: 46.8%; silt content: 16.1%; sand content: 37.1%; water-holding capacity: 55.3%, with clay texture.

The soil moisture was adjusted to 60% of the water-holding capacity (WHC), and 50 g (dry basis) were placed in each respirometer, which was kept in an oven at 28 °C. Prior to the test, the films were cryogenically milled and sieved to obtain granules smaller than 5.0 mm. In this test, microcrystalline cellulose in powder form was used as a positive control. The soil without any additional materials was also tested. The carbon content of the samples was analyzed using a PerkinElmer elemental analyzer, model 2400 Series II. Based on these results, the quantity of each sample to be added to each bottle was determined to achieve 60 mg of carbon, following the recommendation of ASTM D5988.^31^ The test was performed in duplicate.

The Bartha respirometer is illustrated in Figure 1. A volume of 10 mL of a 0.2N potassium hydroxide (KOH) solution was added to the side arm of the respirometer, which was used to trap CO_2_ during the test. This solution was periodically removed from the respirometer and transferred to an Erlenmeyer flask. The side arm of the respirometer was then washed three times with 10 mL of CO_2_-free deionized water, and the volume from the washings was also added to the Erlenmeyer flask. The titration was carried out with 0.1N hydrochloric acid (HCl), using phenolphthalein as the indicator. This procedure was performed once a week for the first 7 weeks and then every 2 weeks until the 180-day period was complete. Based on the initial weight of each respirometer during the monitoring days, the moisture was adjusted by adding water as necessary.

**Figure 1:**
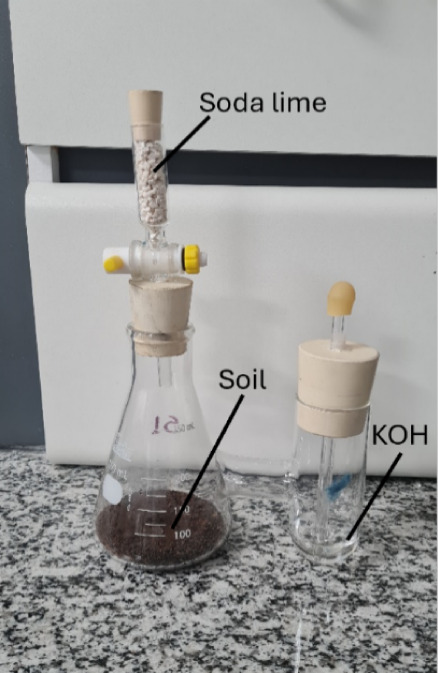
Bartha respirometer.

After each measurement, a fresh KOH solution was added to the respirometer, and compressed air, devoid of CO_2_ and absorbed by soda lime, was injected for 5 min into the upper side of the Erlenmeyer flask to enhance aeration.

The calculation of produced CO_2_ was conducted according to eq 6:

6 $${\mathrm{mg CO}}_{2}=\left(B-V\right)\times N\times E$$

Where:

mg of CO_2_—produced CO_2_ (mg);

*B*—volume of 0.1 N HCl used in the titration for the flask containing only soil;

*V*—volume of 0.1 N HCl used in the titration referring to the respirometer containing soil and samples;

*N*—normality of HCl (0.1 N);

*E*—gram equivalent of CO_2_ = 22.

To calculate the percentage of mineralization, the amount of carbon dioxide produced was divided by the theoretical mass of carbon dioxide and then multiplied by 100. The theoretical value of carbon dioxide was determined by using eq 7:

7 $${\mathrm{mg CO}}_{2}\mathrm{\_t}=\frac{44\times C}{12}$$

Where:

mgCO_2__t—theoretical carbon dioxide mass (mg);

*C*—amount of carbon in the sample (mg);

44—molar mass of carbon dioxide;

12—atomic mass of carbon.

The selected best-performing nanocomposite films and neat PBAT films were also subjected to ecotoxicity analysis using a bioassay with *A. cepa*. First, the milled films (less than 5 mm) were mixed with 500 g of soil (the same as in the biodegradation test) at a concentration of 1% by mass in 2-L glass flasks and kept in an oven at 28 °C for 180 days, alongside the biodegradation test. The soil moisture was adjusted to 60% of water-holding capacity (WHC), and weekly during the test, the moisture was corrected as needed by adding water based on the initial weight of each flask. The flasks were maintained in the oven at 28 °C throughout this period. Before biodegradation, as well as after 3 and 6 months of biodegradation, the soil samples were removed from the oven and stored at 7 °C until the preparation of the aqueous extract, which was then used in the bioassay.

The soil samples were prepared to create an aqueous extract, following the Brazilian Standard NBR 10006.^32^ This involved mixing 100 g (dry basis) of the soil samples, with and without films, with 400 mL of reverse osmosis water. The mixtures underwent mechanical agitation for 5 min and were kept at room temperature for 7 days. After this period, the samples were filtered using a 0.45 mm porosity filtration membrane. These samples were identified with suffixes corresponding to the biodegradation periods: before (T0), after 3 months (T3M), and after 6 months (T6M), respectively.

Bioassays using *A. cepa* were conducted following a protocol proposed by Grant,^33^ with some modifications. Seeds of the Diamantina (Isla) were utilized. In each Petri dish, 100 seeds were placed on a filter paper. Three Petri dishes were prepared for each sample. Then, 6 mL of the aqueous extract was added to each plate. Reverse osmosis water was used as the negative control (NC). The positive control (PC) consisted of a 4 × 10^–4^ M methylmethanesulfonate (MMS) solution. The Petri dishes were maintained at 22 °C with a photoperiod of 12 h of light and 12 h of darkness. After 5 days, the roots were collected and fixed in a mixture of ethanol and acetic acid (3:1 v/v) for 6 h at room temperature. After this period, the ethanol/acetic acid mixture was replaced with a freshly prepared one. The roots were stored at 4 °C until slide preparation.

The fixed roots underwent the Feulgen reaction.^34^ The meristems and F1 region were placed on the slides with a drop of acetic carmine (2%) and covered with coverslips. The material was gently crushed, and the coverslips were removed by using liquid nitrogen. Permanent slides were made with a synthetic resin (Entelan).

The analysis of slides was performed in a light microscope using a 40× objective. Around 5000 cells were counted per treatment in each region (meristematic and F1), with 500 cells counted per slide and 10 slides evaluated for each treatment. The quantified parameters in the meristematic region were Mitotic Index—MI (given by the percentage of cells in division) and Chromosomal Alterations Index—CAI (given by the percentage of cell alterations). Besides, in both meristematic and F1 regions, the micronucleus—MN percentage was also quantified.

Statistical analysis was conducted using GraphPad Prism 10 software, applying the D’Agostino and Pearson test for normality and the Kruskal–Wallis test (with a 95% confidence level), followed by Dunn’s multiple comparisons to NC.

## 3 Results and Discussion

### 3.1 Characterization of the PBAT and Nanocomposite Films

The molecular weights (*M*) of PBAT (pellets), neat PBAT film, and nanocomposites are presented in Table 2.

**Table 2:** Molecular Weight, Thickness, and UV/Vis Transmittance of PBAT and Nanocomposite Films

Sample	*M* (g/mol)	Thickness (μm)	UV (300–400 nm)/Vis (400–800 nm) Transmittance (%)
PBAT (pellets)	134 526	-	-
PBAT^a^	119 820	51 ± 2	UV: 35.7 ± 0.5
			Vis: 87.3 ± 0.4
			Total: 71.3 ± 0.5
PBAT/GO0.25^a^	92 075	59 ± 2	UV: 25.8 ± 0.0
			Vis: 75.3 ± 0.2
			Total: 60.0 ± 0.1
PBAT/GO0.50	51 590	64 ± 4	UV: 19.4 ± 2.1
			Vis: 63.5 ± 3.7
			Total: 49.9 ± 3.2
PBAT/CB1.5	132 178	55 ± 3	UV: 0.006 ± 0.001
			Vis: 0.056 ± 0.001
			Total: 0.041 ± 0.001
PBAT/GO0.25CB1.5	101 164	51 ± 2	UV: 0.006 ± 0.001
			Vis: 0.028 ± 0.005
			Total: 0.021 ± 0.003
PBAT/GO0.50CB1.5	55 820	65 ± 2	UV: 0.007 ± 0.001
			Vis: 0.005 ± 0.001
			Total: 0.0056 ± 0.0002

a Cardoso et al.^35^

It is possible to observe that the incorporation of GO primarily affected the reduction of molecular weight (*M*) during processing by extrusion, likely due to hydrolysis resulting from the water molecules adsorbed between GO sheets.^36^ The degradation is much more pronounced in the presence of GO at 0.5% (for both formulations, with and without carbon black). Regarding the PBAT pellets, the sample with only 0.25% of GO experienced a 32% reduction in *M*, which was lessened in the presence of carbon black (PBAT/GO0.25CB1.5), which suffered a reduction of 25%. The influence of carbon black on this property is evident in the formulation with only this filler (PBAT/CB1.5), which did not experience a reduction in *M*. This behavior can be attributed to specific interactions between water and the oxidized surface of carbon black, reducing the available water involved in hydrolysis during extrusion processing.^37^

The thickness and UV/vis total transmittance of the obtained films are presented in Table 2. Figure 2 illustrates the visual appearance of these films. An increase in the GO content led to a decrease in the films’ transparency and UV/vis total transmittance. With a higher GO content (0.5%), compared to neat PBAT film, there was a 46% reduction in UV. GO can act to protect PBAT from photodegradation, which is a highly desirable characteristic for mulch films.^2^

**Figure 2:**
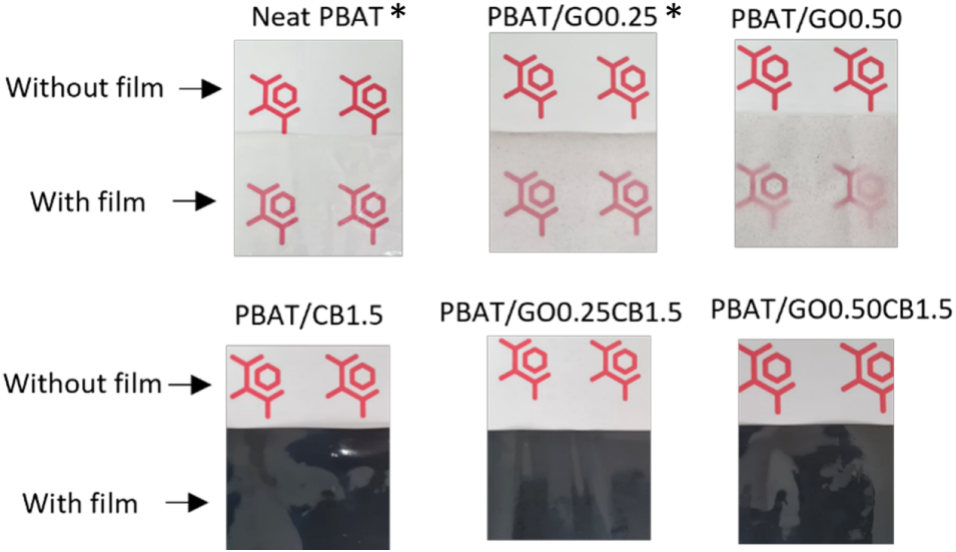
Visual aspects of the produced films. Reprinted in part from Cardoso et al.^35^ ouzalvesechine Licensed under CC BY 4.0.

In the other formulations, it can be observed that carbon black is mainly responsible for a significant reduction in both UV/vis transmittance and a change in the visual appearance of the film, which becomes opaque (black color). Carbon black is a typical UV absorber used to prevent PBAT photodegradation.^7^ The total transmittance for all films was below 0.1%, meeting the European Standard EN 17033 criteria for mulching films, which is less than 3%.^38^

XRD patterns are shown in Figure 3a. The neat PBAT film exhibited peaks at 17.7°, 21.2°, and 23.3°, corresponding to crystalline planes (010), (110), and (100), respectively. Shoulders at 16.2°, 20.2°, and 24.8° were also present, corresponding to crystalline planes (011), (101), and (111).^39,40^ Similar patterns were observed in nanocomposite films, but with higher intensity, primarily associated with plane (101), likely due to the formation of crystalline regions perpendicular to this plane.^41^ The degree of crystallinity remained unaffected after the insertion of GO and CB into the PBAT matrix, and the results for this parameter were found to be within a narrow range (6.0–7.6%).

**Figure 3:**
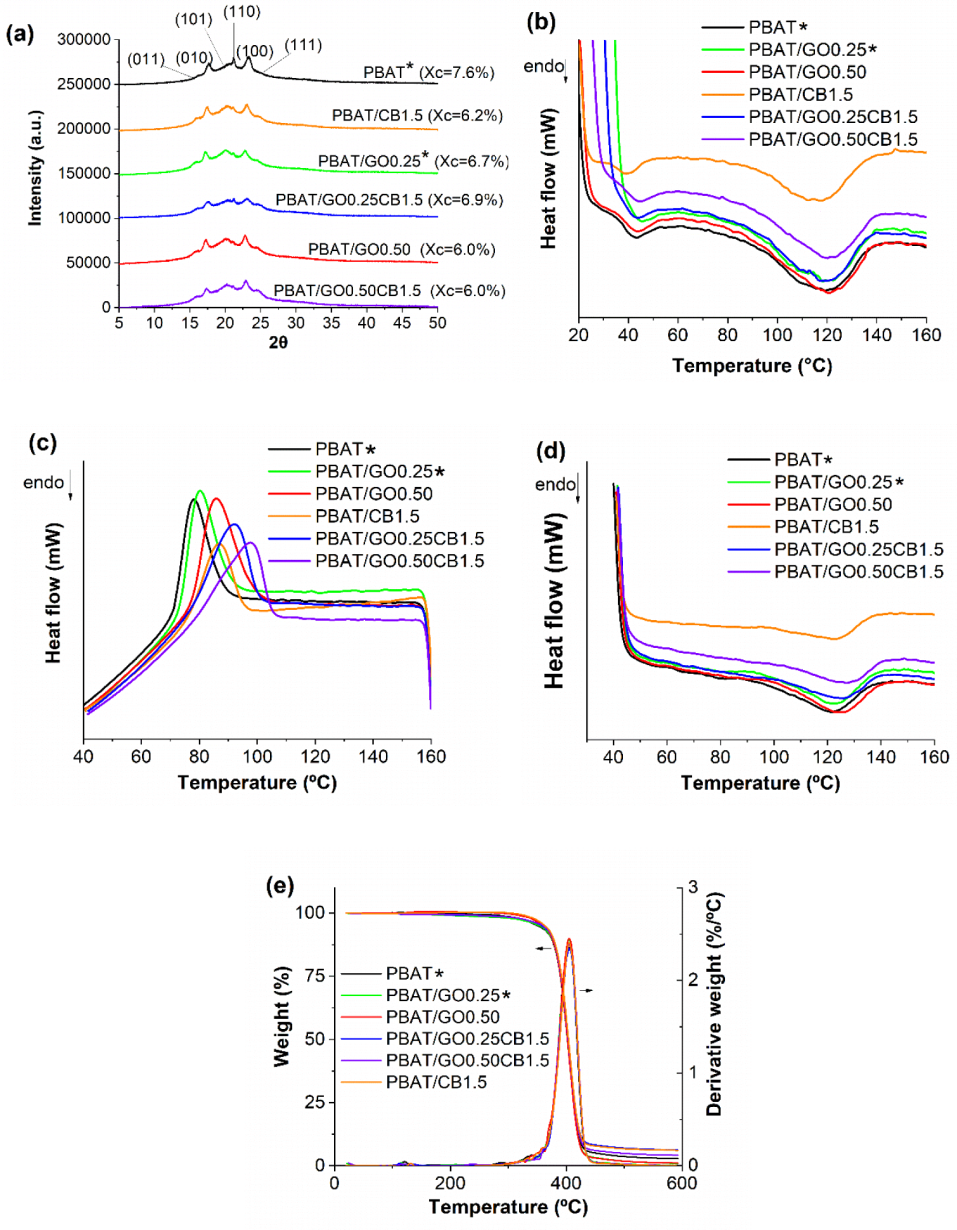
(a) XRD patterns of PBAT and nanocomposites with GO and CB; (b) first heating DSC curves; (c) cooling DSC curves; (d) second heating DSC curves; (e) TGA and DTG curves. *Reprinted in part from Cardoso et al.^35^ Licensed under CC BY 4.0.

Figure 3 shows the DSC curves for different cycles (first heating—Figure 3b, cooling—Figure 3c, and second heating—Figure 3d), and Table 3 presents the results from the DSC analysis. During the first heating, an endothermic peak was identified for all films, with temperatures ranging from 39.1 to 46.6 °C. This peak can be related to the PBAT melting temperature of crystal domains in the aliphatic region (butylene adipate). The broader peak at higher temperatures (116.5 to 120.6 °C) can be associated with the melting of crystal domains in the aromatic region (butylene terephthalate).^42^ In both the first and second heating, the degree of crystallinity did not vary significantly.

**Table 3:** DSC and TGA Results for PBAT and Nanocomposite Films

Samples	DSC—First heating				DSC—Cooling		DSC—Second heating			TGA	
	*T*m_1_ (°C)	*T*m_2_ (°C)	Δ*H*_m_ (J/g)	*X*_c_ (%)	*T*_c_ (°C)	Δ ***H*_c_** (J/g)	*T*_m_ (°C)	Δ*H*_m_ (J/g)	*X*_c_ (%)	*T*_5%_ (°C)	*T*_max_ (°C)
PBAT^a^	46.5	116.5	13.7	12.0	77.6	14.9	121.1	9.1	8.0	350.7	404.8
PBAT/GO0.25^a^	45.8	118.2	17.4	15.3	80.2	16.8	121.6	9.7	8.5	353.8	404.2
PBAT/GO0.50	43.7	120.6	16.3	14.3	85.9	18	125.9	8.9	7.8	359.4	404.7
PBAT/CB1.5	39.1	117.4	16.7	14.7	86.9	12.5	121.8	6.5	5.8	363.0	404.9
PBAT/GO0.25CB1.5	43.8	117.5	16	14	92.2	15.6	125.1	6.7	6.0	357.5	405.5
PBAT/GO0.50CB1.5	44.4	120.1	16.4	14.4	97.7	15.9	127.1	6.6	5.9	360.1	405.0

a Cardoso Cardoso et al.^35^

It was observed that, in the second heating, the crystallization degree increased with the incorporation of GO and CB, despite the rise in *T*_c_. This is likely due to the strong interactions formed between the particles and the PBAT polymer chains, which restrict the mobility of the chains.^43^ Such limitation can interfere with the orderly arrangement of the chains, resulting in a decrease in the crystalline phase, as shown by the lower *X*_c_ values in the nanocomposites. Similar behavior was reported in PHBV/GO^44^ and polyamide 1010/GO^45^ systems.

Considering the cooling cycle, all the nanocomposite films exhibited a higher crystallization temperature value compared to PBAT (*T*_c_ = 77.6 °C), increasing with filler content, from 80.2 °C (PBAT/GO0.25) to 97.7 °C (PBAT/GO0.50CB1.5). This indicates the nucleating effect of both GO and CB^46^ on PBAT, which was also confirmed by XRD analysis.

Figure 3e presents thermogravimetric curves and their derivatives for the produced films, while Table 3 shows the results for *T*_5%_ (the temperature associated with 5% weight loss) and *T*_max_ (the maximum mass loss decomposition temperature). The *T*_max_ results were comparable across the different formulations, whereas *T*_5%_ indicated improvements in the thermal properties of nanocomposites compared to neat PBAT. The inclusion of GO in PBAT nanocomposites may enhance thermal properties, as GO’s impermeable flakes can restrict the movement of decomposition products.^47^ Conversely, carbon black acts as a potent radical scavenger and can trap radicals generated during PBAT decomposition, forming a cross-linked network that makes it more challenging for the decomposition products to escape.^48^

Figure 4a–f shows an optical microscope image of the produced films. Unlike neat PBAT, all the nanocomposites exhibited microagglomerates. The surface area quantified for these agglomerates is indicated in Figure 4g. The average results for this parameter were as follows: 388.1 μm^2^ (PBAT/GO0.25),^35^ 426.9 μm^2^ (PBAT/GO0.50), 500.7 μm^2^ (PBAT/CB1.5), 400.5 μm^2^ (PBAT/GO0.25CB1.5), and the highest value of 1066.3 μm^2^ obtained for the formulation with the highest concentration of fillers (PBAT/GO0.50CB1.5). The increase in GO content led to agglomerates with higher surface area, but without significantly affecting the average. The presence of carbon black only increased the agglomerate’s surface area for the formulation with 0.5% GO, probably due to the high concentration of total additives (2% in weight) and poor dispersion. On the other hand, adding carbon black to the formulation with 0.25% GO did not affect this parameter, indicating that better dispersion was achieved in this case.

**Figure 4:**
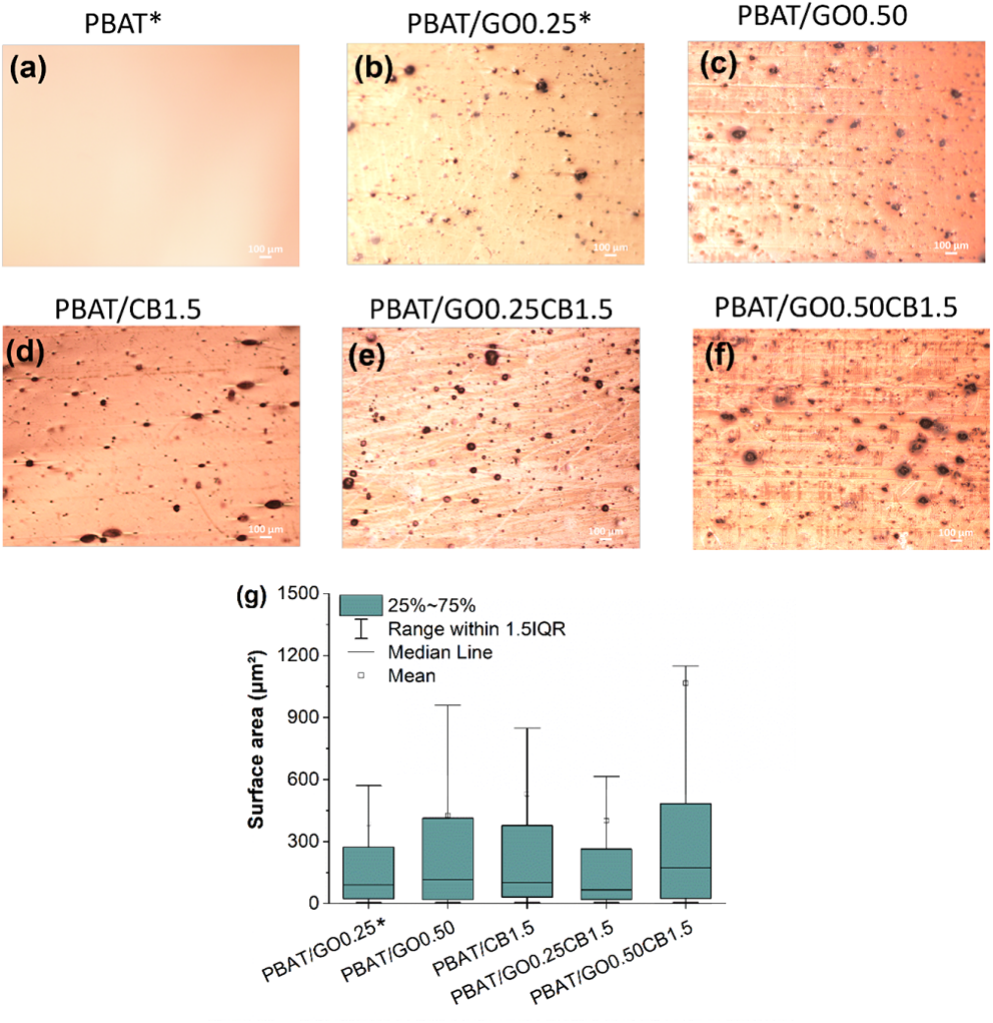
Optical microscopy analysis: (a) PBAT; (b) PBAT/GO0.25; (c) PBAT/GO0.50; (d) PBAT/CB1.5; (e) PBAT/GO0.25CB1.5; (f) PBAT/GO0.50CB1.5; (g) Boxplot graph—surface area of agglomerates. *Reprinted in part from Cardoso et al.^35^ Licensed under CC BY 4.0.

These characteristics can alter the mechanical properties of the films, as discussed later.

Figure 5 presents SEM-FEG microscopy images of the films, which allowed us to observe that as a consequence of the incorporation of fillers, PBAT fracture surface became rougher in the nanocomposites. As reported in our previous work,^35^ in the image related to the formulation PBAT/GO0.25, a GO microagglomerate was verified, and no voids or defects were identified in its surroundings, indicating good adhesion between PBAT and GO.^49^

**Figure 5:**
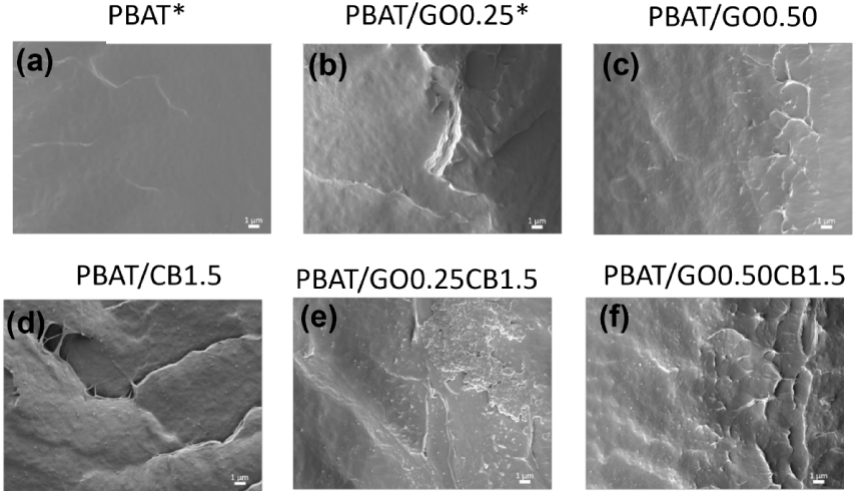
SEM-FEG micrographs of PBAT and nanocomposite films (magnification 5000×): (a) PBAT; (b) PBAT/GO/0.25; (c) PBAT/GO0.50; (d) PBAT/CB1.5; (e) PBAT/GO0.25CB1.5; (f) PBAT/GO0.50CB1.5. *Reprinted in part from Cardoso et al.^35^ Licensed under CC BY 4.0.

Figure 6a shows stress–strain curves obtained from tensile tests. Table 4 presents the results of the Young’s modulus, elongation, tensile strength, and tenacity (area under the curve).

**Figure 6:**
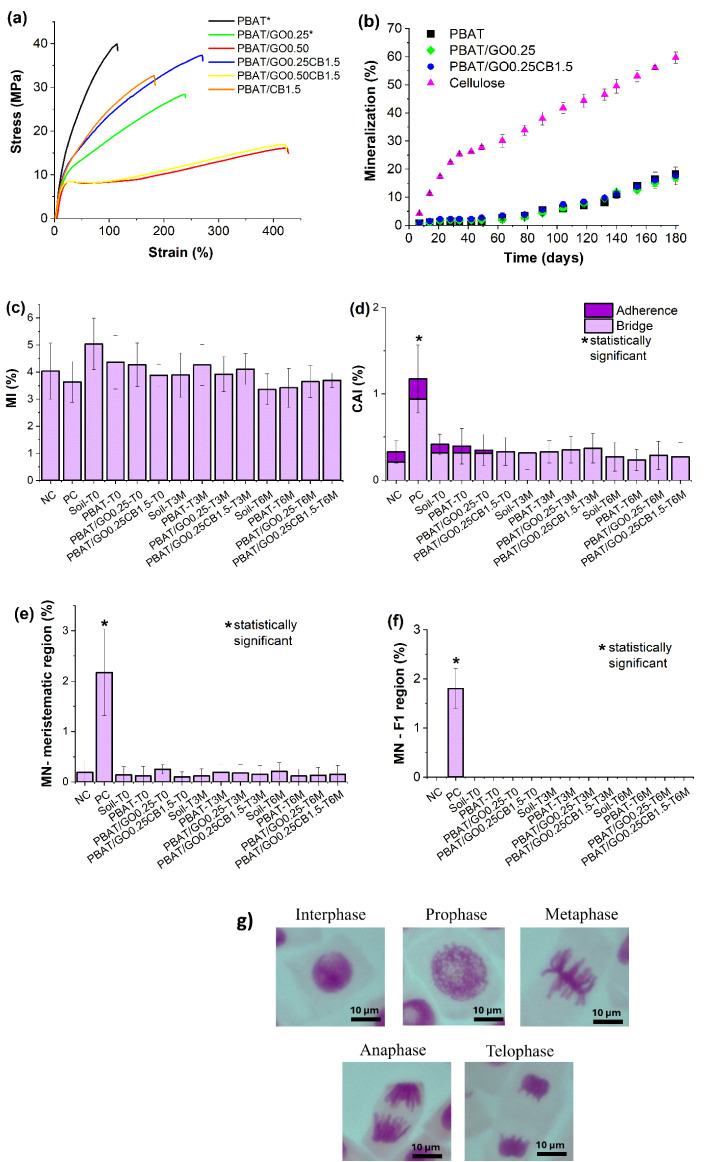
(a) Stress × strain curves for PBAT and nanocomposite films (*Adapted from Cardoso et al.^35^ Licensed under CC BY 4.0; (b) mineralization curves; (c) mitotic index before and after 3/6 months of biodegradation; (d) chromosomal alterations index before and after 3/6 months of biodegradation; (e) micronucleus at the meristematic region before and after 3/6 months of biodegradation; (f) micronucleus at F1 region before and after 3/6 months of biodegradation; (g) meristematic cells of *A. cepa* in interphase and different phases of division at normal condition.

**Table 4:** Mechanical Properties of PBAT and Nanocomposite Films

Samples	Young’s modulus (MPa)	Strain at break (%)	Stress at break (MPa)	Tenacity (10^3^kJ/m^3^)	OP (Barrer)
PBAT^a^	83 ± 4^a^	127.7 ± 21.3	39.0 ± 4.6	35 ± 10^a^	0.92 ± 0.00
**PBAT/GO0.25** ^a^	78 ± 5^a^	242.8 ± 33.2	27.8 ± 1.7	46 ± 10^a^	0.80 ± 0.01
**PBAT/GO0.50**	75 ± 3	411.8 ± 15.8	15.8 ± 0.6	46 ± 2	0.74 ± 0.02
**PBAT/CB1.5**	79 ± 3	192.2 ± 4.2	32.5 ± 0.5	43 ± 1	0.95 ± 0.01
**PBAT/GO0.25CB1.5**	85 ± 4	254.3 ± 26.3	36.1 ± 1.9	63 ± 9	0.80 ± 0.04
**PBAT/GO0.50CB1.5**	76 ± 2	427.8 ± 21.9	17.3 ± 1.0	51 ± 5	0.75 ± 0.01

a CardosoCardoso et al.^35^

The Young’s modulus was the least affected mechanical property, displaying a narrow range of results among the samples (74.8–84.6 MPa). A correlation can be observed between mechanical properties and molecular weight (*M*), as indicated in Table 2, where lower values of *M* result in reduced tensile strength and increased elongation. Probably, hydrolysis during processing can form shorter polymer chains, which can diminish polymer entanglements, thereby reducing polymer strength and increasing its extensibility.^50^ As described before, this process occurred in the presence of GO at 0.25 wt %, and it was intensified at a higher GO concentration (0.50 wt %). In the case of formulation PBAT/CB1.5, which exhibited a similar molecular weight to neat PBAT, the increase in elongation can be attributed to the relative motion (slippage) of the chains at the filler interface under strain.^49^

The presence of GO agglomerates, verified by optical microscopy, could also contribute to the reduction of tensile strength as the concentration of this filler increases from 0.25 to 0.50%. When combined with carbon black, the formulation with lower GO content demonstrated an improvement in this property, which was attributed to the contribution of CB in restoring this property, as a similar tensile strength to neat PBAT was observed in the formulation with only CB. The improvement occurs even with the molecular weight reduction of 16% observed for this film (PBAT/GO0.25CB1.5) compared to neat PBAT. It shows a higher tensile strength than the formulation that did not experience a reduction in molecular weight (PBAT/CB1.5), reinforcing a synergistic effect between carbon black and graphene oxide.

On the other hand, for the formulation PBAT/GO0.50CB1.5, this improvement was not observed, and a reduction in tensile strength of approximately 56% occurred, likely due to the larger surface area of agglomerates in this composition and the abrupt decline in molecular weight.

In addition to tensile strength, the synergistic effect is also evident when evaluating tenacity. 46 470.8 kJ/m^3^ was recorded for PBAT/GO0.25, and 43 065.8 kJ/m^3^ was recorded for PBAT/CB1.5. However, when considering the combination of fillers in the PBAT/GO0.25CB1.5 film, this property reached a value of 63 413.3 kJ/m^3^, indicating an increase of 82% compared to neat PBAT.

Valentini et al. observed a synergistic effect between carbon black (CB) and graphene nanoplatelets (GNP) in an EPDM matrix. The authors proposed that CB primarily formed aggregates on the surface of GNP, linking the gap distance between GNPs and resulting in increased interfacial resistance in the hybrid composite.^51^

Considering the European requirements for the elongation and tensile strength of agricultural films, which are 200% and 18 MPa,^38^ only two of the formulations would be deemed adequate for this application: PBAT/GO0.25 and PBAT/GO0.25CB1.5. The film with a GO content of 0.25% would not meet the criteria for light transmission, falling below 3% for use as a mulch film. Nevertheless, since it exhibited properties that block UV radiation (vital for delaying PBAT photodegradation) without compromising the visible transmittance of PBAT, it has the potential for use as greenhouse films. This application requires transmittance in the visible wavelength (400–800 nm), which is essential for photosynthesis and plant growth.^52,53^

Table 4 presents the oxygen permeability coefficient calculated for PBAT and nanocomposite films. The quantified permeability oxygen coefficient (OP) for neat PBAT was 2385.0 ± 9.9 cm^3^·mil/m^2^ ·d·atm (or 0.92 Barrer), consistent with the value reported in the literature of 2440 cm^3^·mil/m^2^ ·d·atm.^54^ This property is an important indicator of the filler morphology and dispersion. As noted, graphene oxide (GO) is the filler that contributes to a reduction in OP, which is expected since it can create a tortuous path for gas diffusion.^55^ The formulation containing only carbon black (PBAT/CB1.5) showed a similar OP result to neat PBAT, which is anticipated since this filler is spherical and has a low aspect ratio compared to graphene derivatives.^56,57^

Figure 6b presents the mineralization results, with the highest values obtained for cellulose, which is expected since it is used as a positive control in the test. The level of biodegradation achieved during the experiment for neat PBAT in this study (18.2%) aligns with findings from other studies. Souza et al.^58^ observed mineralization in soil of 18%, and Palsikowski et al.^59^ verified a result of 16%, both over a period of 180 days.

Considering the formulations, the behavior related to biodegradation was very similar among them, and after 180 days, a mineralization degree of 18.2 ± 2.5% was achieved for neat PBAT, 16.9 ± 2.5% for PBAT/GO0.25, and 17.6 ± 0.4% for PBAT/GO0.25CB1.5. Therefore, the results indicate that none of the fillers compromise PBAT biodegradability in soil.

Figure 6c–f presents the results obtained from the *A. cepa* bioassay: Mitotic Index (MI), Chromosomal Alterations Index (CAI), and Micronucleus (MN) in meristematic cells and F1 region. Regarding the MI results, none of the tested samples showed a statistically significant difference compared to the negative control (NC). The positive control (PC), MMS, was the only sample with a statistically significant difference in the results of the parameters CAI and MN in both the meristematic and F1 regions, which is consistent since MMS is a known genotoxic and mutagenic substance for this test organism.^60,61^ Figure 6g illustrates *A. cepa* meristematic cells at interphase and in different phases of division (prophase, metaphase, anaphase, and telophase), under normal conditions, which was the predominant pattern observed in the soil samples associated with PBAT and its nanocomposites.

Generally, the main chromosomal alterations observed in the different treatments were chromosomal bridges and adherences. However, considering the statistical analysis before and after biodegradation (3 and 6 months), none of the studied formulations exhibited genotoxic effects on *A. cepa*. Additionally, the absence of cytotoxic effects was confirmed by MI results, while the absence of genotoxic effects was evidenced by MN analysis in the meristematic region, and the absence of mutagenic effects was evident through MN analysis in the F1 region. This lack of toxicity across various parameters was also noted by Souza et al.^10^ for PBAT films with and without carbon black combined with phenolic and amine ultraviolet stabilizers. However, it is important to emphasize that in that study, the authors used a lower initial concentration of films in soil (0.2%) for toxicity evaluation with *A. cepa*. In the present study, an initial concentration of films in soil of 1% was employed, following the recommendations of the European Standard EN 17033,^38^ as this allows for simulating the concentration at various thicknesses and considering the potential for multiple applications of films in soil. The absence of toxicity is a crucial indicator of the promising application of the tested films in agriculture.

## 4 Conclusions

The goal of this work was successfully achieved, allowing for the verification that both graphene oxide and carbon black are promising for enabling PBAT agricultural films. Our results indicate that the optimal GO content in formulations for achieving these improvements is 0.25%. Based on the synergistic effect of carbon black and graphene oxide observed for tenacity, it is possible to consider the formulation PBAT/GO0.25CB1.5 as the most suitable for application as a mulch film.

In recent years, the production of graphene and its derivatives has shifted toward companies with high manufacturing capacity while maintaining material quality. This change has lowered the prices, making these materials more viable as additives in polymer matrices. The results shown here demonstrate the scientific feasibility of using not only graphene oxide (GO) but also its combination with carbon black for agricultural films. Implementing this product does not require changes to current film production lines, as it uses the same equipment used in conventional agricultural film manufacturing-only replacing the polymer matrix with a biodegradable material and adding specific nanofillers. Ultimately, market demand and global sustainability goals will drive the adoption of this product in the future, aligning its performance with ecological and economic benefits.

Although the formulation PBAT/GO0.25 also presented adequate mechanical properties for mulch application, its high transmittance (60%) would not prevent the weed growth due to the high level of photosynthetically active radiation passing through the film, and it is not recommended for this application. Nevertheless, it could be applied in cases where transmittance is desired, such as in greenhouse films.

The fact that PBAT biodegradability was not affected by the presence of GO and GO in combination with carbon black combined with the fact that the formulations did not present cytotoxic, genotoxic, and mutagenic effects to *A. cepa*, reinforces the promising alternatives of these nanocomposites for agriculture as a possibility to reduce residue production caused by the use of conventional films for mulching and greenhouses.

Considering our results, the absence of toxic effects for *A. cepa* occurs during 6 months of degradation. Since the formation of polymer byproducts can occur throughout the entire period before complete mineralization, long-term ecotoxicity tests are recommended. Since *A. cepa* is a solid bioassay with high sensitivity at a chromosomal level, the results suggest a promising application for PBAT nanocomposites with carbon black and graphene oxide in agricultural films, considering not only their material properties but also their environmental safety. In future work, we plan to explore other bioassays, such as acute toxicity tests on plants, earthworms, and nitrification inhibition tests with soil microorganisms, as recommended by the European Standard EN 17033/2018 for certification purposes.
